# Patients' views on responsibility for the management of musculoskeletal disorders – A qualitative study

**DOI:** 10.1186/1471-2474-10-103

**Published:** 2009-08-17

**Authors:** Maria EH Larsson, Lena A Nordholm, Ingbritt Öhrn

**Affiliations:** 1 Department of Clinical Neuroscience and Rehabilitation/Physiotherapy, Institute of Neuroscience and Physiology, The Sahlgrenska Academy, University of Gothenburg, Sweden; 2 The Vårdalinstitutet – The Swedish Institute for Health Science, Lund University, Sweden; 3 Research and Development Unit, Primary Care, County of Gothenburg, Sweden; 4 University College of Borås, Sweden; 5 Institute of Health and Care Sciences, The Sahlgrenska Academy, University of Gothenburg, Sweden

## Abstract

**Background:**

Musculoskeletal disorders are very common and almost inevitable in an individual's lifetime. Enabling self-management and allowing the individual to take responsibility for care is stated as desired in the management of these disorders, but this may be asking more than people can generally manage. A willingness among people to take responsibility for musculoskeletal disorders and not place responsibility out of their hands or on employers but to be shared with medical professionals has been shown. The aim of the present study was to describe how people with musculoskeletal disorders think and reason regarding responsibility for prevention, treatment and management of the disorder.

**Methods:**

Individual interviews with a strategic sample of 20 individuals with musculoskeletal disorders were performed. The interviews were tape-recorded, transcribed verbatim and analysed according to qualitative content analysis.

**Results:**

From the interviews an overarching theme was identified: own responsibility needs to be met. The analysis revealed six interrelated categories: Taking on responsibility, Ambiguity about responsibility, Collaborating responsibility, Complying with recommendations, Disclaiming responsibility, and Responsibility irrelevant. These categories described different thoughts and reasoning regarding the responsibility for managing musculoskeletal disorders. Generally the responsibility for prevention of musculoskeletal disorders was described to lie primarily on society/authorities as they have knowledge of what to prevent and how to prevent it. When musculoskeletal disorders have occurred, health care should provide fast accessibility, diagnosis, prognosis and support for recovery. For long-term management, the individuals themselves are responsible for making the most out of life despite disorders.

**Conclusion:**

No matter what the expressions of responsibility for musculoskeletal disorders are, own responsibility needs to be met by society, health care, employers and family in an appropriate way, with as much or as little of the "right type" of support needed, based on the individual's expectations.

## Background

Musculoskeletal disorders are very common and almost inevitable in an individual's lifetime. Lifetime prevalence for low back pain is for example 58–84% and the point prevalence, i.e. proportion of people suffering from back pain at a particular point of time, is 4–33% [1]. Musculoskeletal disorders are also a common reason for self-medication and entry to the health care system [2]. The impact of musculoskeletal conditions has been recognized and a task force of the Bone and Joint Decade (2000–2010) has among other things provided a standards of care document for acute and chronic musculoskeletal pain [3]. In this document a rigorous review and summary is made of documents concerning management of musculoskeletal conditions produced over the last years. Enabling self-management and allowing the individual to take responsibility for care is stated as desired in the management of the disorders.

In a thesis by Kjellström [4], called Responsibility, Health and the Individual, concepts of individual responsibility for health were studied. The study showed that the prerequisites for taking responsibility were self-reflection, critical examination and conscious choices. She also examined concepts about personal responsibility for health from the perspective of development theory and concluded that some demands require more than people can generally manage.

Larsson and Nordholm [5] presented a cross-sectional study on attitudes of responsibility for managing musculoskeletal disorders. It was shown that a majority of participants had an internal view regarding responsibility of managing musculoskeletal disorders, i.e. they thought that they should take responsibility themselves and did not place responsibility out of their hands or on employers to any great extent. A shared responsibility for musculoskeletal disorders between the individual and medical professionals was also indicated. Associations were found regarding attitudes towards responsibility for musculoskeletal disorders and background variables; mainly to physical inactivity, musculoskeletal disorder related sick leave and to no education beyond compulsory level, which increased the odds of attributing responsibility externally, i.e. placing responsibility on someone or something else.

To meet or influence the attitudes regarding musculoskeletal disorders, it is necessary to also know more about how people think and reason regarding this; why and on what grounds do they place responsibility mostly on themselves or on someone or something else? There is a need to know more about the underlying thoughts and reasoning for the taken attitude.

The aim of the present study was to describe how people with musculoskeletal disorders think and reason regarding responsibility for prevention, treatment and management of the disorder.

## Methods

### Informants and procedure

A strategic sample [6] of 20 individuals with musculoskeletal disorders was recruited via physiotherapy outpatient clinics in the county of Southern Bohuslän (Sweden) for interviews to obtain individual experiences and perceptions. The inclusion criteria were having or had musculoskeletal disorder primarily generated by the musculoskeletal system, over 18 years of age and Swedish speaking. After a verbal request to participate from their physiotherapist they were given a letter with information about the study. If they were interested in participating in an interview, their physiotherapist provided the researcher with their name and phone number. The researcher then contacted them by phone with a request to participate in the study. Before the interview started, the informants were again informed about the study and told they could withdraw from the study at any time. They were also given opportunity to pose questions. The informants then gave their verbal informed consent and were assured confidentiality. The note with name and phone number was destroyed after the interview which could subsequently not be connected to the informant's identity. None of the informants declined or discontinued participation in the study. Three pilot interviews were conducted in October 2007, 17 interviews were conducted in April to July 2008. As the pilot interviews did not differ significantly they were included in the study. The last six interviews did not seem to provide much new information and the data collection was therefore ceased. The Regional ethical review board in Gothenburg was consulted prior to the study, and formal ethical approval was deemed unnecessary according to Swedish law.

According to the informant's choice, 17 interviews took place at a location near the physiotherapy department where they had been patients; one interview was performed at the university where the interviewer worked, one at the informant's workplace and one was a telephone interview. Three of the individuals were immigrants, one from another Nordic country, one from the Baltic States and one from the Middle East. Semi structured interviews were performed with each of the informants individually. An interview guide was used. To gain information about thoughts and reasoning regarding responsibility for the musculoskeletal disorder, the informants were asked to narrate their recent experience of musculoskeletal disorders. The first question was followed by open questions about how the informants thought the disorder started, their beliefs about the cause of the disorder, their treatment experience, to whom they turned for help and why and their management and prevention of the disorders. In the latter part of the interview, the informants were also explicitly asked about their thoughts and reasoning about responsibility for prevention, treatment and management regarding the disorder.

The interviews were tape-recorded, mean interview time was 42 minutes (19 min – 1 h 18 min), and then transcribed verbatim by the first author.

### Analysis method

To meet the aim of the current study, a qualitative content analysis was used [7]. The unit of analysis was the text transcribed from the interviews. Each interview was read several times, bearing in mind the aim of the study, in order to get a sense of the content. An inductive approach was taken in the analysis. The data were systematically analysed for meaning units, which were subsequently condensed and then coded [7]. Throughout these stages the analysis stayed close to the data [7,8]. When the whole material was coded a categorisation procedure started. Sub-categories were formed through a group of codes with content that shared a commonality; identified by the thread throughout the codes. The sub-categories were then sorted and abstracted into categories. Finally the underlying meaning, the latent content of the categories was formulated into a theme [7]. Nvivo 8 software (QSR International Pty Ltd), was used to analyse the interviews and quotations.

The third author, with a different occupation (nurse), more experienced in the method but less experienced in the field, read the transcripts of interviews and checked codes and categories performed by the first author and the codes and categories were discussed until consensus was reached. Examples of meaning units, condensed meaning units, subcategories, categories and theme are shown in Additional file 1.

## Results

Eleven women and nine men participated in the study. Mean age was 52.3 years (range 25–78 years), six had compulsory education, nine high-school and five university education. Eight had been on sick-leave at some point for a shorter or longer time during the last three months. The individuals were generally at the end of, or had finished their physiotherapy treatment period. Three individuals had or previously had low back pain, four had back pain in combination with disorders from upper or lower extremities, four had disorders from upper extremities and one in combination with knee problems, three had pain from lower extremities, and five had multiple site musculoskeletal disorders. The cause of the disorder was described as being of importance for the management of musculoskeletal disorders. Generally, if the disorder appeared without explanation or due to own actions, the responsibility to manage the disorder was placed on oneself. However, if the disorder was due to external factors, responsibility for the management was generally placed externally.

From the interviews an overarching theme was identified: Own responsibility needs to be met. The analysis revealed six interrelated categories about responsibility for managing musculoskeletal disorders: **Taking on responsibility**, **Ambiguity about responsibility**, **Collaborating responsibility**, **Complying with recommendations**, **Disclaiming responsibility, Responsibility irrelevant.**

The theme (capital letters), categories (bold type) and their subcategories (italics) are presented below in the text. Codes from one interview may be present in several categories and the categories may have representation from one or more interviews. Table 1 shows percentages and number () of how codes of each interview are distributed over the categories. Table 2 shows the interviews'/informants' representation in each of the categories.

**Table 1:** Percentages and number () of codes in each interview distributed over the categories.

	**Taking on responsibility**	**Ambiguity about responsibility**	**Collaborating responsibility**	**Complying with recommendations**	**Disclaiming responsibility**	**Responsibility irrelevant**
**Interview 1**	35% (7)	0	10% (2)	0	40% (8)	15% (3)

**Interview 2**	44% (14)	3% (1)	22% (7)	25% (8)	0	6% (2)

**Interview 3**	28% (8)	21% (6)	10% (3)	0	24% (7)	17% (5)

**Interview 4**	29% (10)	3% (1)	18% (6)	15% (5)	29% (10)	6% (2)

**Interview 5**	54% (15)	0	7% (2)	0	25% (7)	14% (4)

**Interview 6**	40% (24)	0	10% (6)	10% (6)	35% (21)	5% (3)

**Interview 7**	48% (22)	0	26% (12)	22% (1)	17% (8)	7% (3)

**Interview 8**	42% (18)	0	19% (8)	2% (1)	28% (12)	9% (4)

**Interview 9**	35% (22)	13% (8)	21% (13)	19% (12)	6% (4)	6% (4)

**Interview 10**	40% (22)	2% (1)	18% (10)	27% (15)	4% (2)	9% (5)

**Interview 11**	14% (5)	0	6% (2)	9% (3)	54% (19)	17% (6)

**Interview 12**	24% (21)	2% (2)	41% (35)	21% (18)	7% (6)	5% (4)

**Interview 13**	40% (19)	8% (4)	6% (3)	13% (6)	33% (16)	0

**Interview 14**	26% (11)	2% (1)	36% (15)	10% (4)	26% (11)	0

**Interview 15**	49% (18)	0	16% (6)	11% (4)	24% (9)	0

**Interview 16**	40% (12)	0	27% (8)	3% (1)	23% (7)	7% (2)

**Interview 17**	36% (12)	0	9% (3)	42% (14)	12% (4)	0

**Interview 18**	52% (24)	4% (2)	26% (12)	9% (4)	7% (3)	2% (1)

**Interview 19**	34% (14)	0	29% (12)	24% (10)	10% (4)	2% (1)

**Interview 20**	38% (18)	0	19% (9)	12% (6)	15% (7)	17% (8)

For example, in Interview 1 there were no codes matching the category of "Complying with recommendations" while Informant 2 (Interview 2) had 25% of her codes in that category (% not always ends up to 100% due to round up).

**Table 2:** The Interviews' (informants') representation in each of the categories.

	**I1**	**I2**	**I3**	**I4**	**I5**	**I6**	**I7**	**I8**	**I9**	**I10**	**I11**	**I12**	**I13**	**I14**	**I15**	**I16**	**I17**	**I18**	**I19**	**I20**
**Taking on responsibility**	X	X	X	X	X	X	X	X	X	X	X	X	X	X	X	X	X	X	X	X

**Ambiguous about responsibility**		X	X	X					X	X		X	X	X				X		

**Collaborating responsibility**	X	X	X	X	X	X	X	X	X	X	X	X	X	X	X	X	X	X	X	X

**Complying with recommendations**		X		X		X	X	X	X	X	X	X	X	X	X	X	X	X	X	X

**Disclaiming responsibility**	X		X	X	X	X	X	X	X	X	X	X	X	X	X	X	X	X	X	X

**Responsibility irrelevant**	X	X	X	X	X	X	X	X	X	X	X	X				X		X	X	X

***A core story***[9,10] was then formulated based on the categories and the theme in order to illuminate thoughts and reasoning about responsibility for the prevention, treatment and management of musculoskeletal disorders.

### OWN RESPONSIBILITY NEEDS TO BE MET

No matter what the expressions of responsibility are, own responsibility needs to be met by society, health care, employers and family, met in an appropriate way, with as much or as little of the "right type" of support as needed, based on the individuals' expectations.

#### **Taking on responsibility**

Taking on responsibility means that no matter what disorders a person might have, they are his or her responsibility. The informants described that it was only they themselves who can take on responsibility for the disorders. The participants described it to be their own responsibility to seek help from health care and to be persistent in getting the help they need. Taking on responsibility also means to be persistent in adhering to treatment and taking responsibility for the result. Self-treatment is also used. Physical activity is seen as beneficial for mental well-being and there is a belief that disorders are managed more effectively if you are physically fit. The participants stressed that good self-knowledge is important in the management of the disorders and different strategies can be used to balance the disorders in life. The prevention of recurrences was expressed as being your personal responsibility.

##### *Ending up with me anyhow*

Informants expressed that there is an individual responsibility and that one can't place burden for the disorder on anyone else. "Who but me would take care of myself?" They reasoned that it is your own life and your own body, and responsibility for well-being lies with oneself. Participants described that it is up to the individual to use information and to take measures. They expressed that you can not rely on anyone else to solve things for you. In the end, they reason, it is your own responsibility to manage the disorder, as you have to live with it and face the consequences of failing to do so.

##### *Seeking expertise*

The informants described that the responsibility for the disorder is mainly their own, that help could be needed from those with more knowledge in the matter, generally health care professionals. In most cases the informants had tried to manage their disorder on their own but when the condition worsened or became recurrent they sought help. It could be that the disorder interferes with work or life too much. Generally, they find it to be their own responsibility to seek help in time. For some of the informants it was a significant undertaking to seek help and it could be hard to know who to contact for help. The importance of getting help when needed from medical professionals and that they really listen and believe in them is stressed. The participants believe that medical professionals can help with advice, exercise or medication but that without self-responsibility no expertise in the world would help.

##### *Persistency*

A great deal of persistency is needed as described by the informants. They described the necessity of being persistent to get the treatment they required. Some informants felt they had to be very mentally alert to get what they need and that it is to a great extent left to the individual to get the care needed. The informants described that persistency is also needed regarding the disorder itself. Many have tried "quick fixes" by for example a chiropractor but realized that this would not work long term without doing more about the disorder themselves. They expressed that one have to "hang in there" with recommended exercise. Being patient and having a routine in treatment was described as necessary to recover and overcome restrictions.

##### *Self-treatment*

Self-treating strategies are described, such as adjusting posture or position at work. A number of times the interviewees did not seek any help for the disorder, but used self-treatment with a device and/or non-prescriptive medication. Some also expressed that they wanted to try without medication and sometimes invented treatment on their own when nothing else helped.

##### *Perform physical activity to enhance well-being*

The informants stated that they performed physical activity as it felt good for both body and soul, which in turn made better pain management possible. Exercise is seen as necessary for mental well-being and disorders can be better handled when the person is spry and fit.

##### *Self knowledge for use of managing strategies (avoiding, balancing, accepting and secondary prevention)*

Self-knowledge is considered very important. The informants described that as it is their lived body, they need to learn their own reactions. Despite medical expertise, they expressed that you know your own body best and what works for you. Self-perception helps to manage the disorders. The informants described how they take on own responsibility by the use of management strategies. It could be by anticipating and *avoiding *disorder triggering movements or activities which could keep the disorder under control. However, the fear of pain could also lead to restrictions in activities. Another strategy is to *balance *life to reduce disorders. Balancing life is achieved by listening to the body, taking things at your own pace and putting reasonable strain on the body. Some of the informants expressed a desire to live a more unhurried life, which would probably affect the disorder. Some informants expressed that a form of *acceptance *is achieved. They describe how they have accepted having disorders and learnt to live with them and manage them as well as possible. It could be to do alternative things or in an alternative or compensative way. The informants described that they re-evaluated what is important and renegotiated the starting-point of the disorder. Having had a disorder and knowing it could be recurrent should lead to taking measures for *secondary prevention*, reasoned many of the informants. It could be in terms of thinking more about how to use your body, taking ergonomic measures, exercising to strengthen muscles to be better prepared for your workload, all in order to avoid future disorders. The interviewees expressed that they also need to consider alternative ways of doing things and need to know what their limits are.

#### **Ambiguity about responsibility**

Ambiguity in the reasoning about responsibility for musculoskeletal disorders means that on one hand the informants think it is their own responsibility to manage musculoskeletal disorders but on the other hand they feel that work demands precede the management. Ambiguity can also include knowing what to do but not doing it.

##### *Work demands precede management*

Some informants expressed that they consider managing the disorder at work as their responsibility but find it difficult, due to pressure on production. Others felt that easier and less stressful work could have prevented the disorder, and that work demands are superior to the well-being of the employees. Many said that they knew the employer has formal employer responsibility but were unsure to what extent.

##### *Knowing but not doing*

Knowing what to do, be willing and motivated to do so, but still not taking measures to manage the disorder is also described by the interviewees. Even if they are motivated it can be hard for example to fit exercises into daily life. They described acceptance of personal responsibility but at the same time, a need for "sticks and carrots" as own perspective is sometimes too narrow.

#### **Collaborating responsibility**

Collaborating responsibility means that the responsibility for managing musculoskeletal disorders is a collaborative process with others. There is a societal responsibility to keep people active and working but also a responsibility for the individual as a member of society to look after themselves. Society should also provide accessibility for better self-care. Health care must provide necessary prerequisites, correct referral processes and availability, as it is essential for management of the disorders. Involvement from the workplace is stressed as important for prevention of musculoskeletal disorders as well as in the management when disorders have occurred. Support from the family is also important.

##### *Need for keeping people active and in work*

A societal responsibility to keep citizens active and working was expressed by some of the informants. It was mentioned both as health promotion and for economic reasons as sick-leave uses tax money and means which costs society. Some informants would like the social insurance system to be more flexible, take more responsibility in rehabilitation and invest more in the individual, but also set demands on the individual as with unemployment benefits. Another suggestion is that there should be economic support from society for ergonomic measures in the workplace.

##### *Accessibility needed*

The informants expressed that society could provide better possibilities for physical activity and self-care as well as facilities and expertise at a lower cost to people who are trying to manage their disorder outside the health care system. Society is seen to have a responsibility to provide accessibility and support to people who want to manage musculoskeletal disorders.

##### *Prerequisites to manage needed*

To handle the disorder the informants stressed the importance of health care providing diagnosis and prognosis for the disorder. When health care fulfil their obligations by providing this, it gives the informants prerequisites to act upon and a collaborative process can take place. Some informants thought that health care underestimated the importance of providing diagnosis and prognosis which can obstruct rehabilitation.

##### *Referral processes needed*

The informants expressed that they think there is a medical responsibility to ensure a proper examination is carried out to determine what you are suffering from. If the medical professional doesn't know what it is or can't help, it is his/her responsibility to ensure a referral. Referral could be to another medical specialist, to specific examinations, to operation or to other medical professions such as physiotherapists. The informants thought there should be a medical responsibility for referral. Lack of referral in the patient's opinion, leads to disappointment and causes a delay in correct treatment. Self-referral is sometimes used. Physicians are seen to have power as gate-keepers.

##### *Availability needed*

Health care is often thought to be too bureaucratic, availability is lacking and the informants complained of lack of understanding from health care professionals concerning need for prompt availability. The informants expected health care to provide technical aid if needed, support instead of questioning, provide better information about possible treatments and provide the treatment the doctor prescribed. Informants experienced that health care sometimes does not fulfil their promises and obligations and they believe that more resources should have been made available, but at the same time recognise the matter of limited resources as well. Availability and back up is expressed to be essential to manage the disorder. Ultimately the politicians are seen to be responsible for availability.

##### *Workplace involvement needed*

The informants expressed a need for companies and employers as well as company doctors to be involved in the management of musculoskeletal disorders. Company health policies with investments in personnel are needed. The informants believed that employers should be responsible for education and prevention as with ergonomic measures, provide facilities for and support exercise, but also show involvement and empathy when employees have a disorder. Informants also expressed the view that the employer is responsible for the workplace but it is their own responsibility to follow advice and safety precautions and to ensure they perform work in a safe manner. If the disorder is work-related the employer's responsibility for rehabilitation and prevention of reoccurrence is emphasised.

##### *Emotional support needed*

A need for emotional support from everyone around you, employer and colleagues as well as family is mentioned as important by the informants when suffering from musculoskeletal disorders. The surrounding factors in collaboration influence disorder management.

#### **Complying with recommendations (as a way of adopting responsibility)**

Complying with recommendations means adopting responsibility by actively following advice and recommendations. Society should give information, teach ergonomics and provide information about as well as give opportunities for physical activity. Health care should provide guidance for politicians as well as for the general public on how to best manage musculoskeletal disorders. Once a disorder is present, the individual is obligated to carry out what is recommended, to persist in getting relief from the disorder and not give up. There is also a parental responsibility to ensure that children follow advice for a healthy lifestyle.

##### *Provision of information, ergonomics, exercise needed*

The informants expressed that the responsibility for prevention of musculoskeletal disorders is greater for society than for the individual. Society has knowledge about what needs to be prevented and how this can be done, and should provide this. Then it is the responsibility of the individuals to follow recommendations. The informants also expressed a belief in physical activity and muscular strengthening as prevention of musculoskeletal disorders and an understanding that the body needs maintenance. Learning ergonomics is also believed to prevent disorders. For both exercise and ergonomics, the informants think school is an excellent platform, both to provide information but also to set a practical standard. They expressed that learning early in life could be a preventative measure. Informants expressed that societal prevention and elimination of musculoskeletal disorders should be seen as an investment.

##### *Provision of guidance needed*

Health care and medical professionals have to convey the benefits of physical activity as a preventive measure and a health investment to politicians as well as to the general public, as expressed by the informants. Health care should also provide guidance for fun and motivating exercise to promote joy as well as prevention. Even maternal care should provide information and exercises for back disorder prevention.

##### *Carrying out recommended advice or treatment*

The informants described how they have sought help from medical professionals as they need professional support and need to know that they are doing "the right thing". They did not have any specific request of treatment but a wish for good results. They don't consider there to be many alternatives other than to follow the advice given by health professionals. They may seek advice if there are problems but don't dare to exercise on their own or seek alternative medicine. Some described how they follow the advice of medical professionals, on for example an exercise program, but state that otherwise they have no interest in performing exercise. Some also expressed the need for a firm hand as they may be lacking in self-discipline but realise that effort has to be made to reach results.

A wish for an independent adviser who could give the perfect advice to follow was considered the optimal way to manage the disorder.

##### *Parental need of support for healthy lifestyle*

Some informants expressed that parents should make sure their children live a healthy lifestyle. They believe that parents are responsible for passing on knowledge and support of a healthy lifestyle by providing opportunities and conditions as well as by setting good examples as role models. Thus a healthy lifestyle comes naturally.

##### *Keep trying to get relief from disorder*

The informants expressed that they have to follow recommendations and cannot give up and feel sorry for themselves. They described how they might need to accept the seriousness of the condition and that rehabilitation could take time. The informants expressed that one must keep trying with all means available, try everything to get relief from the disorder and recover.

#### **Disclaiming responsibility**

This category expresses the idea that management of musculoskeletal disorders consists of receiving treatment. Help and treatment to resolve the disorder are expected to be given and the individual does not adopt own responsibility. Medical professionals have a responsibility to manage the disorders as they have the knowledge and education and informants put great confidence in them doing so. The medical professionals are thereby also responsible for the result of treatment and if recovery defaults, disappointment with the professionals sometimes occurs.

##### *Be given help/treatment*

The informants described receiving treatment. They expressed how they turn to someone to receive treatment, not so much to get help with what to do themselves, but to be given help/treatment to alleviate the pain or resolve the disorder. A "quick fix" is often preferred. Sometimes the informants described how they have tried a treatment recommended by an acquaintance or one which has helped before. The informants stated it is important to receive the right medication, aids, X-ray or sometimes an operation is the only solution. Some described how physiotherapy was provided when needed or how they manage their disorder with regular visits to a, for example, chiropractor or naprapath. They also explained that the characteristics of symptoms determine who you turn to with the disorder and if they have tried the available treatments, they have at least tried some alternatives. Some informants expressed that they had high hopes about improvement and high expectations on medical care. A certain disappointment is sometimes expressed with medication or treatment when it only represses symptoms or gives temporary relief, but some find it a great relief to get help and don't believe in full recovery.

##### *Relying on professionals with knowledge to act*

The informants expressed that the primary responsibility lies with the doctors to provide advice, medication or other treatment. They believe the medical professionals take responsibility, as the patients cannot have knowledge about the disorder or the recovery. Seeing the "right" medical professional is considered important. Some of the informants expressed that different types of disorders require different types of medical professionals who are then responsible for communicating with each other in the best interest of the patient. The medical professionals should also decide how much the patient can work. Medical check-ups might be needed to keep the disorder under control. Some also expressed that if they have managed to get the treatment they believe is necessary (such as an operation) they have done what they can; the rest is up to the professionals. Many informants show great confidence in medical professionals; physicians and physiotherapists as well as naprapaths, osteopaths and chiropractors. They expect medical professionals to make them well. However, some also expressed disappointment when promised recovery defaults. The informants think that medical professionals sometimes neglect to take necessary actions and believe that the professionals should have known the appropriate treatment needed.

#### **Responsibility irrelevant**

When responsibility is seen as irrelevant it stands for the belief that musculoskeletal disorders are due to biological processes such as hereditary and wear and tear of the body. Some people have a predisposition for musculoskeletal disorder, others don't. It is not possible to prevent disorders as it is not possible to know what type of disorders you may sustain.

##### *Biological processes*

It is expressed by the informants that it is not possible to adopt any responsibility and you cannot affect the disorder as the disorder is due to heritage or biological processes in the body. Some people just have a predisposition for disorders. It is believed to be a genetic or a heretical cause. The informants expressed that some people get disorders and some don't, you have to accept and manage your own predispositions. Possibly you might postpone but not prevent the disorder. The biological processes cannot be stopped and recovery is difficult if the body is worn-out.

##### *Unpredictable*

The informants stated that if they don't have any disorders they don't think about prevention for future disorders. How would you know what to do? They can't see how they could have done anything to prevent the disorder. Some expressed that they never tried to avoid or prevent anything; you have to do what you have to do. They believe that disorders caused by accidents cannot be prevented and there are other disorder triggering factors that are not preventable. Many informants expressed the difficulty of preventing something they don't know is coming. Not knowing what is causing the disorder makes it even more difficult.

**Core story**

The responsibility for prevention of musculoskeletal disorders lies primarily on society/authorities as they have knowledge of what to prevent and how to prevent it. When musculoskeletal disorders have occurred, health care should provide fast accessibility, diagnosis, prognosis and support for recovery. For long-term management, the individual is responsible for making the most out of it and living as good a life as possible.

**Figure 1:**
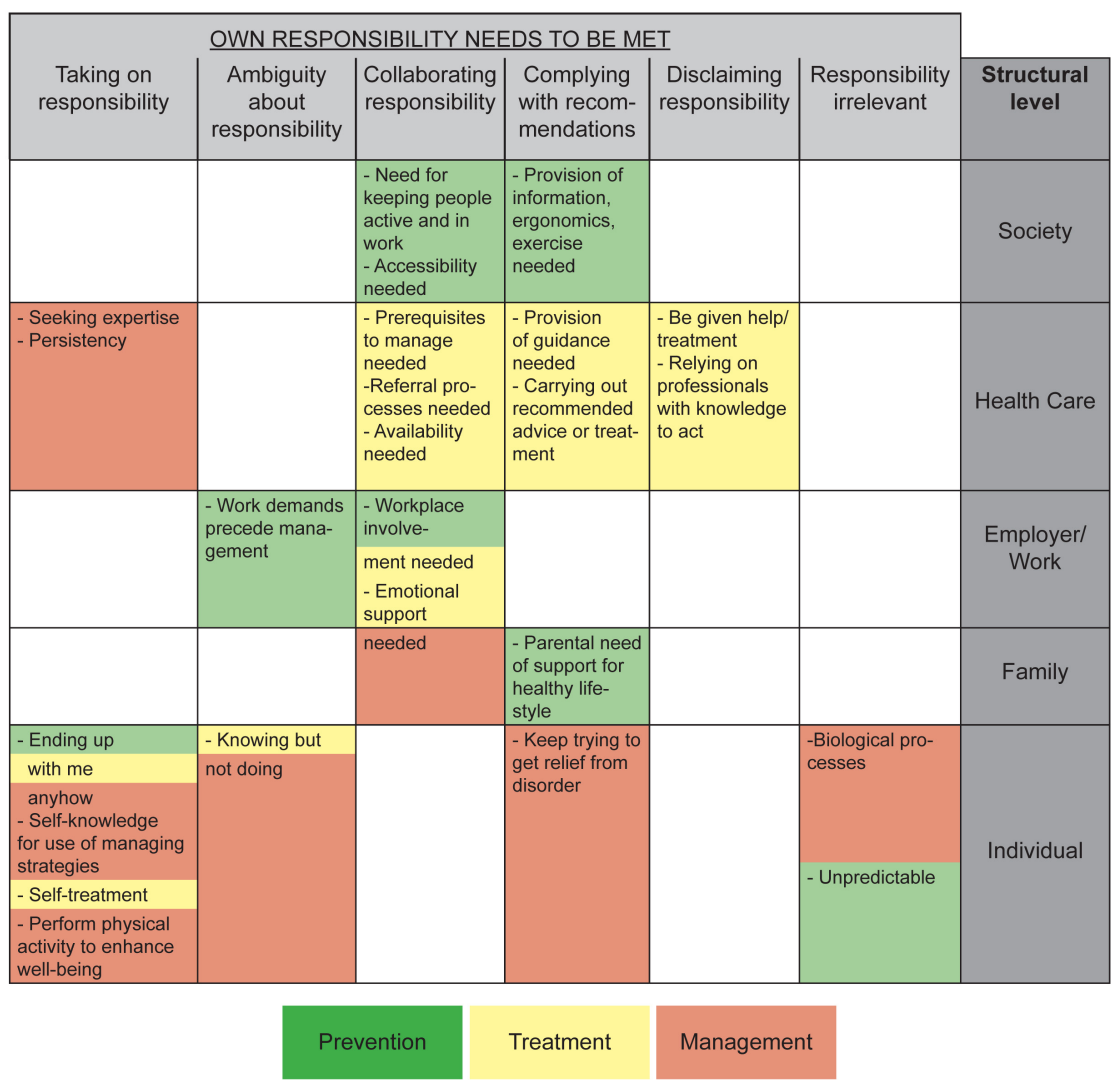
**The interrelationship of categories and subcategories also on a structural level and in relation to prevention, treatment and management**. On the horizontal axis are the categories. On the vertical axis are the structural levels. Subcategories are placed in relation to the category and the structural level they represent and colour-marked with green when the subcategory is expressing prevention, yellow when it is expressing thoughts of treatment and red when the subcategory is expressing management of musculoskeletal disorders.

Figure 1 shows the interrelationship of categories and subcategories also on a structural level and in relation to prevention, treatment and management.

## Discussion

The present study has provided information about how patients with musculoskeletal disorders view responsibility for the management of musculoskeletal disorders.

In the standards of care for acute and chronic musculoskeletal pain document [3], one of the objectives is to promote partnerships among the community, patients and clinicians in decision-making in relation to pain – its prevention and management. Partnership is also stressed as important by the informants in this study. The partnership can however be viewed from different aspects and levels. Partnership can occur on different structural levels such as community, society, health care and employer but the informants also described different views in this partnership. Some informants are without question taking on responsibility for management of the disorder. They believe that it is their responsibility to see to their own body in the best way and to get the right help at the right time. They can't rely on anyone else.

Others see this relationship as a collaborative process in which society, health care and employer and even the family are partners. Yet some express partnership by following what's recommended. The least active partnership for the individual in this matter is to be receiving. No doubt the individuals are experts of their own disorder but there is a need to balance professional knowledge with the individual's expertise. The different standpoints or attitudes taken by the patients require different strategies for health providers for successful management of the disorder.

The self-efficacy concept [11] could be addressed related to the results of the present study. Cognitive, social, emotional and behavioural sub-skills capabilities must be coordinated and organised effectively to serve many purposes. Self-efficacy is not concerned with the number of skills a person has, but with what he believes he can do with what he has [12]. In the category **Taking on responsibility**, a high level of self-efficacy could be seen whereas in **Disclaiming responsibility**, a low level of self-efficacy is shown as the patients want others to manage the disorder. Self-efficacy has been shown to be important in for example rehabilitation after anterior cruciate ligament injury [13].

Taking an active approach towards health management is seen as beneficial. Those who take an active part are more likely to follow treatment regimes. Passive patients may be less likely to have help from others and may be predisposed to sickness to start with. Ironically patients who, on the surface seem to be adjusted or compliant, but are adjusted in a passive way are more likely to be ill at follow up [14].

One might relate the taken attitude of responsibility to coping style. Lazarus's stress and coping model defined coping as "constantly changing cognitive and behavioural efforts to manage specific external and/or internal demands that are appraised as taxing or exceeding the resources of the person" [15]. Two major types of coping were proposed; the problem-focused coping which includes efforts directed at controlling or changing the sources of the stress (here as ways of handling the problem that causes disorders) and emotion-focused coping strategies which are attempts at managing emotional responses to the stressor (strategies for handling for example fears due to the disorder). As coping attempts to diminish the physical, emotional, and psychological burden of the disorder both problem and emotion-focused coping may play a part in the response [15].

Brown and Nicassio [16] further conceptualize coping as active or passive in nature. Active coping was referred to as adaptive strategies used by the individual to control a disorder. On the contrary, passive coping used strategies that gave control of disorder management to others or as acceptance of restrictions in life [16]. Frequent use of passive coping strategies in high pain could contribute to higher levels of depression over time [17]. Taking active responsibility might reduce the risk of disability due to the disorders. A systematic review by Pincus and coworkers [18] in low back pain which investigated cognitive risk factors for disability, passive coping strategies were risk factors for an unfavourable outcome.

The present study's categories **Taking on responsibility**, **Ambiguity about responsibility **and **Collaborating responsibility **could be related to the description of active coping and patients with musculoskeletal disorder might benefit from these taken attitudes for a favourable management. Whilst still taking responsibility but perhaps in a more passive coping way the category **Complying with recommendations **could be seen. The categories **Disclaiming responsibility **and **Responsibility irrelevant **might be seen as related to the possible adoption of passive coping styles.

Another closely related construct and to which the present study's results could be related is internal and external health locus of control, the concept of people having different ways of ascribing responsibility and causality in their lives. This concept was originally developed by Julian Rotter in the 1960s [19] and originally regarded internal and external locus of control of reinforcement, but has been used widely in health-specific instruments such as the Multidimensional Health Locus of Control Scales (MHLC) [20]. Those with an internal locus of control see themselves responsible for the outcomes of their own actions. Someone with an external locus of control sees environmental causes and situational factors as being more important than internal ones.

Larsson and Nordholm [21] further developed these ideas of responsibility to a musculoskeletal specific instrument and in a study of a general population, it was investigated *where *people placed responsibility for musculoskeletal disorder [5]. The present study has explored and described *how *and *why *these attitudes might be taken, how it can be explained, what rationales lay behind a taken attitude. In contribution to what is already known about attitudes and how they might affect management of musculoskeletal disorders, the information provided in the present study could help with how to approach and meet different taken attitudes.

The researchers of this study were not the patients' clinicians nor did they work at the departments from which the informants were recruited (the interviewer (MEHL) worked at one of the departments more than five years ago), so the interviewer was not known to the patients. The interviewer presented herself as a PhD student and did not state medical profession. This approach was used to avoid the interviewer's profession influencing responses. However, some of the informants asked about the interviewer's profession (physiotherapist). Usually this question was posed after the interview but some did ask before the interview started or somewhere in the middle of it. The possibility that this might have affected the responses due to social desirability may not be able to be disregarded. Neither were the patients in the study asked whether they were self-referred or physician-referred to treatment, which might represent differences in their views.

Also, one must reflect on the researchers' possible influence in formulation of the research question, in data collection and of course in the interpretation. The research question was mainly grounded in clinical empirical work but also based on previous studies in the area [5,22]. The data collection was made through strategic sample [6], but it was to a certain degree also a convenient sample by the chosen geographical area. The area is located in the vicinity of Gothenburg (which is the second largest city of Sweden) and includes a mix of both rural and urban districts, with a proportion of people commuting to the metropolitan area but not living there. The issue of generalizability is in qualitative research usually addressed as transferability, which represents the possibility of transferring the results in a study to other settings or groups [23]. It is an empirical matter depending on the similarity between the sending and receiving context. As the present study was performed in Sweden, which still mainly has a socialized health care system although an increasing number of private health providers, one may wonder if informants in for example the U.S. would hold a different view on responsibility? To provide empirical evidence of possibly contextual similarity, descriptions of both the clinical setting and the chosen geographical area were given [7,24]. One can always also discuss whether 20 individuals can be representative for a population, which leads us to the question of confirmability i.e. the degree to which results can be confirmed by others. The results of the present study, have somewhat verified what was found in a study of a general population on how attitudes were placed [5]. But the present sample of 20 individuals did not allow us to make comparisons due to socio-demographic variables which was shown by Larsson and Nordholm in a previous study [5]. Whether the results can be confirmed and are of direct use for the clinic and in other settings or countries still needs to be explored.

One can also speculate about the problem of selection bias of informants as these were recruited by their clinician. Maybe the clinicians recruited the most satisfied patients? This problem was addressed by explicitly explaining to the clinicians that it was not the treatment that was to be evaluated, but patient experiences, with no regard to outcome of treatment.

Working as a physiotherapist for more then ten years will naturally influence beliefs about musculoskeletal disorders and about patients suffering from these disorders even when taking on the role as a researcher. Therefore, it was a strength and advantage for the present study to have two co-researchers from different occupations (nurse, psychologist) and research areas (diabetes, social psychology) when interpreting the results.

Studies have shown that there are disparate attitudes regarding self-responsibility and coping with pain between health care staff and patients where health care staff rated self-responsibility of higher importance for recovery from a work place injury than the patients did [25]. It has also been shown that clinicians might underestimate patients' willingness to take on own responsibility and may overlook an opportunity to promote health [26]. For prevention of recurrent musculoskeletal pain allowing the individual to take responsibility for care with continued support from the family and the physician as well as the employer and other people involved in the process is desired [3].

## Conclusion

The present study has shown different views about responsibility for the management of musculoskeletal disorders. It has provided information on how own responsibility can be taken but also that own responsibility needs to be met by others and suggestions on how this can be done.

It is hoped that these results will help clinicians as well as health planners to understand the views of people who experience musculoskeletal disorders and that different needs must be met depending on different attitudes. Good strategies for the prevention and care of musculoskeletal disorders may then hopefully be developed.

## Competing interests

The authors declare that they have no competing interests.

## Authors' contributions

MEHL involved in conception and design, obtaining grants, development of interview guide, acquisition, performed and transcribed the interviews, preparation of data, analyses and interpretation of data, drafting the article. LAN involved in conception and design, analyses and interpretation of data, substantial contribution in revising the article for important intellectual content. IÖ involved in conception and design, development of interview guide, analyses and interpretation of data, substantial contribution in revising the article for important intellectual content. All authors have read and approved the final manuscript.

## Pre-publication history

The pre-publication history for this paper can be accessed here:



## Supplementary Material

Additional file 1 **Including examples of meaning units, condensed meaning units, subcategories, categories and theme from the qualitative content analysis of patients' views on responsibility for the management of musculoskeletal disorders.** Click here for file
