# Supplementary material for: Patients' views on responsibility for the management of musculoskeletal disorders – A qualitative study
Source: BMC Musculoskelet Disord. 2009 Aug 17;10:103. doi: 10.1186/1471-2474-10-103 (PMC2753559; doi:10.1186/1471-2474-10-103)
Supplement: Additional file 1 — Including examples of meaning units, condensed meaning units, subcategories, categories and theme from the qualitative content analysis of patients' views on responsibility for the management of musculoskeletal disorders. [file 1471-2474-10-103-S1.pdf]

Additional file 1. Examples of meaning units, condensed meaning units, subcategories, categories and theme from the qualitative content analysis of patients' views on responsibility for the management of musculoskeletal disorders.

Meaning unit	Condensed meaning unit	Code	Subcategory	Category	Theme
I feel it when it's back, like I said it's me not anyone else who can, there's noone who can manage my disorder or tell me this is what you should do	Noone else can manage my disorder	Noone else can manage my disorder	*Ending up with me anyhow*	**Taking on responsibility**	OWN RESPONSIBILITY NEEDS TO BE MET
Yes because it's me who has to go, if I want any help, it's me who goes to some doctor or physiotherapist or it's me who has to say what I want. If I don't say anything, then I'm happy with it probably.	It's me who has to say what I want.	My responsibility to ask for help	*Seeking expertise*		
…after which I lived on the phone the whole spring and eventually I ended up….I talked to a lot of hospitals ….	”live on the phone”	Have to be persistent to get the needed treatment	*Persistency*		
I have one of those, … made of rubber that you hit yourself where it really hurts. I've used that, but then I've also used anaesthetic salve or gel …	Uses device and salve	Self treatment with device and medication	*Self-treatment*		
Sometimes I actually exercised and ran regularly as well. Even if I get tired of it pretty quick I usually feel better both physically and mentally.	Feel better physically and mentally through exercise.	Exercise for well-being body and soul	*Perform physical activity to enhance well-being*		
… in the long run, the most important thing is some kind of self-knowledge about how you work….to learn about how the body works and manage it in the best way possible.	Self-knowledge important to manage the disorder.	Self-knowledge most important	*Self knowledge for use of managing strategies*		
…you feel pressure from all directions so it's not that easy …you don't always have time to stop and see how you feel. If you have pain somewhere then someone with the flu takes priority.	Pressure, don't have time to stop and see how you feel.	My responsibility for my disorder but difficult when pressure	*Work demands precede management*	**Ambiguity about responsibility**	
I think an awful lot about the things I should do but that don't get done…and what that's due to, despite the fact that I feel really motivated, it's probably because I don't have enough time or else that's just a bad excuse.	Should do but never get around to it, maybe lack of time.	Thinking and knowing but not doing	*Knowing but not doing*		
Or that I start to cost society money. Well, perhaps I already do (laughs), I just realised, but I mean to be	Cost society money	Societal costs	*Need for keeping people active and*	**Collaborating responsibility**	

on sick leave or something.			*in work*		
Cycle paths for example and nice cross-country tracks where people can run, access to gyms and things like that so people can exercise, but they should be used when they're there.	Access to cycle paths and gyms for people to use.	Societal prerequisites for physical activity	*Accessibility needed*		
It's important to know what it is, that you don't go and wonder what it could be, because when you know about it then you can do something about it and learn.	Important to know what it is, because then you can do something about it and learn.	Diagnosis important, know how to handle	*Prerequisites to manage needed*		
I think I could have been referred to someone who knows what you should do, an orthopaedist or pain clinic or something before it got to this stage, that's what I think. And it isn't that I've just sat at home waiting for something to happen, I've really tried to make it happen.…	Should have been referred before it got to this stage.	Not referring means prolonged disorders (doctor didn't take his responsibility)	*Referral processes needed*		
Yes, I think there is immense bureaucracy around health care, …would it really have mattered…if the emergency room had given me a referral to an orthopaedist?	Immense bureaucracy around health care.	Health care too bureaucratic	*Availability needed*		
You have to take responsibility for yourself … say that it's not working for me now, I'm in a lot of pain but then your place of work has to take responsibility to either let you be at home or at least lessen the workload for the individual.	Own responsibility to speak up, responsibility on place of work to lessen the load.	Must be mutual, from yourself and employer	*Workplace involvement needed*		
There are quite a lot of authorities that just question and I find that difficult. I think that if you feel you have support, you can manage much better and perhaps find alternatives.	Questioning is difficult, can manage better if support is felt	Society should provide support instead of questioning	*Emotional support needed*		
It is, gymnastics a couple of times a week and then learning about your body in some way would have been excellent in school, and then continue in working life as well.	Excellent with gymnastics and learning about your body in school.	School good platform for preventive measures	*Provision of information, ergonomics, exercise needed*	**Complying with recommendations**	
It's the different professional groups who have a responsibility to inform and go out more and lecture and write, all these type of things so that people understand that this is important.	The professionals have a responsibility to inform	Med profs should convey disorder preventive measures	*Provision of guidance needed*		
There's some muscle there on the back there and I	Got acupuncture and	Following treatment	*Carrying out*		

have to exercise so I got acupuncture and some movements to do at home.	exercise at home	program	*recommended advice or treatment*		
If my dad had said no we won’t bother driving to the garage, we’ll walk instead then I might have gone too. If he’d said let’s go swimming, I would have looked at him and said no. But then if he’d said yes we’re going and then we go. If I’d started early then it would become a habit after a while.	Starting early with exercise would have become a habit from home.	Parents see to children’s physical well-being	*Parental need of support for healthy lifestyle*		
And of course you want to be rid of the pain. So then of course you test all available means.	Test all available means to get rid of the pain	Try with all the means at one's command	*Keep trying to get relief from disorder*		
It was a bit like a shortcut if you can put it like that, but it was really convenient, very simple and it was good. Quick and simple it took 15 minutes and it was done.	Like taking a shortcut, fast and simple.	Chiropractor shortcut- quick and simple	*Be given help/treatment*	**Disclaiming responsibility**	
There the responsibility must almost be placed on the women who looked after me, who know how a patient like me should be treated and what to do make the patient well again.	The woman who looked after me knows how it should be treated	Meds profs who have knowledge about the recovery	*Relying on professionals with knowledge to act*		
No I don’t think it can be prevented as it’s hereditary so I don’t think there’s anything….because you can’t do anything about your relatives.	Can’t prevent disorders as they are hereditary	Can't prevent disorder as hereditary	*Biological processes*	**Responsibility irrelevant**	
And why did I get this, that it came so suddenly with my knee, it took me by surprise a little that I hadn’t felt anything like my knee chafing, it came just like that. Had walked one step too many	The problem appeared so suddenly without warning	Disorders just appeared	*Unpredictable*		
